# Reimagining mental health care for newcomer children and families: a qualitative framework analysis of service provider perspectives

**DOI:** 10.1186/s12913-023-09682-3

**Published:** 2023-06-27

**Authors:** Amanda Sim, Afreen Ahmad, Lina Hammad, Yasmine Shalaby, Katholiki Georgiades

**Affiliations:** 1 Department of Psychiatry and Behavioural Neurosciences, McMaster University, Hamilton, ON Canada; 2 Department of Health Research Methods, Evidence, and Impact, McMaster University, Hamilton, ON Canada; 3 Department of Psychiatry and Behavioural Neurosciences, The Offord Centre for Child Studies, McMaster University, Hamilton, ON Canada

**Keywords:** Newcomers, Refugees, Immigrants, Mental Health, Barriers, Service providers, Canada

## Abstract

**Background:**

Persistent disparities in access to mental health care for refugee and immigrant children and their families pose unique challenges to policy and practice. This study examined service provider perspectives on the barriers and opportunities for improving mental health supports for newcomer children and families in Canada.

**Methods:**

Semi-structured individual and group interviews were conducted with 33 leadership and frontline staff from 14 organizations in the health, education, settlement, and social service sectors in Hamilton, Ontario. Interview data were analyzed using the framework method.

**Results:**

Participants described barriers at the systems, provider, and individual and family levels that prevented newcomer families from accessing and benefiting from mental health supports. Structural barriers included inadequate services and funding, complexity of systems, cultural tensions, and, lack of prevention and early identification. Provider-level barriers included lack of representation, mental health knowledge and cultural competency, and staff shortages and burnout. Individual and family-level barriers included lack of mental health literacy, primacy of settlement needs, stigma, fear, and the high threshold for help-seeking. Participants’ recommendations for “reimagining care” related to newcomer engagement, person- and family-centered care, cultural responsiveness, mental health promotion and prevention, workforce diversity and development, collaborative and integrated care, and knowledge generation and uptake.

**Conclusions:**

The intersection of structural, provider, and individual/family-level barriers reduce newcomer families’ access to and effectiveness of mental health supports. Reducing disparities in mental health and access to care will require a paradigm shift in the way that mental health care is conceptualized and delivered to newcomer children and families.

**Supplementary Information:**

The online version contains supplementary material available at 10.1186/s12913-023-09682-3.

## Background

Canada’s population is becoming increasingly diverse with over 1.3 million new immigrants settling in Canada from 2016 to 2021, the highest number of recent immigrants recorded in a Canadian census [1]. The demographic characteristics of new immigrants have shifted significantly over time with Asia and the Middle East now the region of origin for 62% of new immigrants [2]. This shift is in part due to Canada’s support of refugee resettlement efforts, most notably in response to protracted conflicts in the Middle East. From 2016 to 2021, 218,430 refugees were admitted to Canada, of which 27.8% and 7.1% originated from Syria and Iraq respectively [2].

The changing demographics of immigration to Canada pose new challenges to mental health care policy and practice. Research suggests that post-migration mental health outcomes differ by admission category and region of origin, with those arriving as refugees or from the Middle East and West Asia having poorer self-reported mental health compared to economic immigrants and immigrants from the United States and Latin America [3]. Child and adolescent newcomers to Canada are a population of particular concern due to their potential exposure to pre-, peri-, and post-migration stressors during a critical period of development. Numerous studies link risk factors such as war exposure and acculturation stressors to poor mental health outcomes among refugee and immigrant children and adolescents, including those resettled in high-income countries such as Canada [4]. For example, research with children and adolescents from Syria and Iraq has found high levels of exposure to potentially traumatic events which in turn were associated with elevated depression, anxiety, and post-traumatic stress symptoms [5, 6]. Visible minority refugee and immigrant children may also experience increased risk of mental heath difficulties due to racism and discrimination related to their religion (e.g. Islamophobia) and/or visible minority status [7]. Despite these well-documented risks, studies consistently find underutilization of mental health services among refugee and immigrant children and youth compared to Canadian-born peers with similar mental health status [8, 9]. These persistent disparities in service access may contribute to the decline in mental health seen over length of stay and successive generations of immigrant children in Canada [10, 11].

Studies conducted in Canada point to a combination of systemic/structural, provider, and individual factors that influence mental health service use among immigrants and refugees, including availability of services, workforce diversity, and stigma [12–14]. While some of these factors constrain mental health care access for the Canadian population at large, others highlight the unique barriers experienced by refugee and immigrant families – particularly those who are racialized or from visible minority groups – and the need for tailored strategies to improve access to care [15].

The 2012 Mental Health Strategy for Canada emphasizes the need to increase diverse communities’ access to services [16]. Although providers in the health, settlement, education, and social service sectors play a crucial role in identifying refugee and immigrant child and youth mental health difficulties and facilitating access to care, Canadian evidence on provider experiences and perspectives remains scarce [13, 17]. Recent socio-cultural shifts such as the changing demographics of immigration to Canada and growing awareness of systemic racism have changed the landscape of mental health need and service provision, further underscoring calls for research on improving mental health equity [18]. The current study aims to address these evidence gaps by examining barriers and opportunities for improving mental health care for newcomer children and families from the perspectives of providers in a range of service delivery contexts. Specific research objectives are to: (1) examine service provider perspectives on barriers to access and effectiveness of mental health supports for newcomer children and families; and (2) identify opportunities and recommendations for improving newcomer mental health supports. In this study, we refer to all refugees and immigrants who have settled in Canada within the past five years (i.e., 2016–2021) as newcomers.

## Methods

### Study design and setting

The study used a qualitative framework analysis methodology consisting of semi-structured individual and group interviews with leadership and frontline staff from the health, education, settlement, and social service sectors in Hamilton, Ontario. Hamilton is the third largest city in Ontario with a population of approximately 582,000 [2]. It ranks fifth nationally and second in Ontario for its proportion of foreign-born population at 24.1% [1]. According to the 2016 and 2021 census, Arabic is now the most commonly-spoken language in the home (after English) in Hamilton [1].

### Participants

The study used a combination of purposive and snowball sampling to recruit leadership and frontline staff from organizations that serve newcomer children and families across a wide range of health, education, and social service sectors. Potential participants were identified by an advisory group established for this study representing refugee and immigrant-serving organizations and municipal government agencies in Hamilton. The study team made initial contact with potential participants by email to introduce the study and provide an information sheet and informed consent form. Participants indicated consent by signing and returning the consent form and were also asked to suggest individuals from their own or other organizations who may be interested in the study. We determined that no further sampling was required when (a) representation from leadership and frontline staff in the health, education, settlement, and other social service sectors was achieved; and (b) no new themes were emerging from the interviews as determined during weekly debriefing meetings among the research team.

### Data collection

A semi-structured interview guide was used to facilitate 24 individual and 4 group interviews with a total of 33 study participants from January to July 2022. The interview guide was reviewed by the study’s advisory group and included questions on existing mental health supports for newcomer children and youth, barriers to access and effectiveness, and recommendations for improving mental health care. The interview guide focused on Arabic-speaking newcomers who had arrived within the past five years given the size of this language group in Hamilton and their likely exposure to pre- and post-migration adversity. During data collection, however, participants noted that that their responses were relevant to newcomers in general rather than specific to Arabic-speaking newcomers only. Hence, the research team adapted the interviews to give participants the flexibility to respond in relation to newcomers from different ethnocultural and language groups. Interviews were conducted virtually on Zoom by four interviewers with lived experience of immigration to Canada under the supervision of the first author, a qualitative and mixed methods researcher with 15 years of experience conducting research with refugee and immigrant populations. Interviews lasted approximately 60–90 min and were audio recorded for verbatim transcription using the transcription platform Otter.ai. Interviewers reviewed transcripts for accuracy and removed identifying information prior to analysis.

### Analysis

Data analysis followed the 7 stages of the framework method: transcription, familiarization with the interview, coding, developing a working analytical framework, applying the analytical framework, charting data into the framework matrix, and interpreting the data [19]. The framework method sits under the broad umbrella of thematic analysis and aims to identify similarities, differences, and relationships in qualitative data that are ultimately developed into themes [20]. For the current study, two transcripts were independently coded by AS, AA, and LH after transcribing and reviewing the interviews. Codes generated from this exercise were used to develop a first draft of the analytical framework, which was then applied by the group to two additional transcripts. Discrepancies in coding were discussed and adjustments made to the analytical framework as needed. The revised analytical framework was then used to code all remaining transcripts using the qualitative analysis platform Dedoose [21]. Finally, similar codes were grouped into categories which were used to create the framework matrix, summarize data for each category in the matrix (i.e., charting), and develop themes and sub-themes that were best supported by the data. Throughout this process, analytic memos were used to capture initial impressions, reflections, and potential themes emerging from the data. To validate findings, we conducted member checking with advisory group members, many of whom had also participated in the study, by presenting and discussing their feedback on the themes and interpretations of the data [22].

This manuscript was written in accordance with the Standards for Reporting Qualitative Research [23]. The study was approved by the Hamilton Integrated Research Ethics Board (HiREB) and the research ethics committees of participating school boards.

## Results

In total, 16 leadership and 17 frontline staff from 14 organizations participated in the study. Sample characteristics are summarized in Table 1. Most respondents (87.9%) were female, 55% were first-generation immigrants (i.e., born outside Canada), and 60% identified as racialized (i.e., not White or Caucasian). On average, respondents had 14 years of experience working on refugee/immigrant or health equity issues. Participants worked in a range of sectors, including settlement, child and youth services, health, and education.

**Table 1:** Sample characteristics

		Overall sample (n = 33)	Leadership (n = 16)	Frontline (n = 17)
**Gender (% female**, ***n*)**		87.9, *29*	81.25, *13*	94.1, *16*
**Race/ethnicity (%**, ***n*)**				
	White (Caucasian)	39.4, *13*	62.5, *10*	17.6, *3*
	Arab/West Asian	24.2, *8*	18.8, *3*	29.4, *5*
	South Asian	12.1, *4*	6.3, *1*	17.6, *3*
	Black	12.1, *4*	12.5, *2*	11.8, *2*
	Multiple	6.1, *2*	0	11.8, *2*
	East Asian	3.0, *1*	0	5.9, *1*
	Latina	3.0, *1*	0	5.9, *1*
**Born in Canada (%**, ***n*)**		45.5, *15*	56.3, *9*	35.3, *6*
**Years of experience (mean)**		14.0	17.0	11.1
**Sector (%**, ***n*)**				
	Settlement	33.3, *11*	25.0, *4*	41.2, *7*
	Youth Development	15.2, *5*	6.3, *1*	23.5, *4*
	Mental Health	15.2, *5*	18.8, *3*	11.8, *2*
	Public/government	15.2, *5*	31.3, *5*	0
	Education	9.1, *3*	0	17.6, *3*
	Ethnocultural	6.7, *2*	6.3, *1*	7.1, *1*
	Community Health	6.7, *2*	12.5, *2*	0

**Fig. 1:**
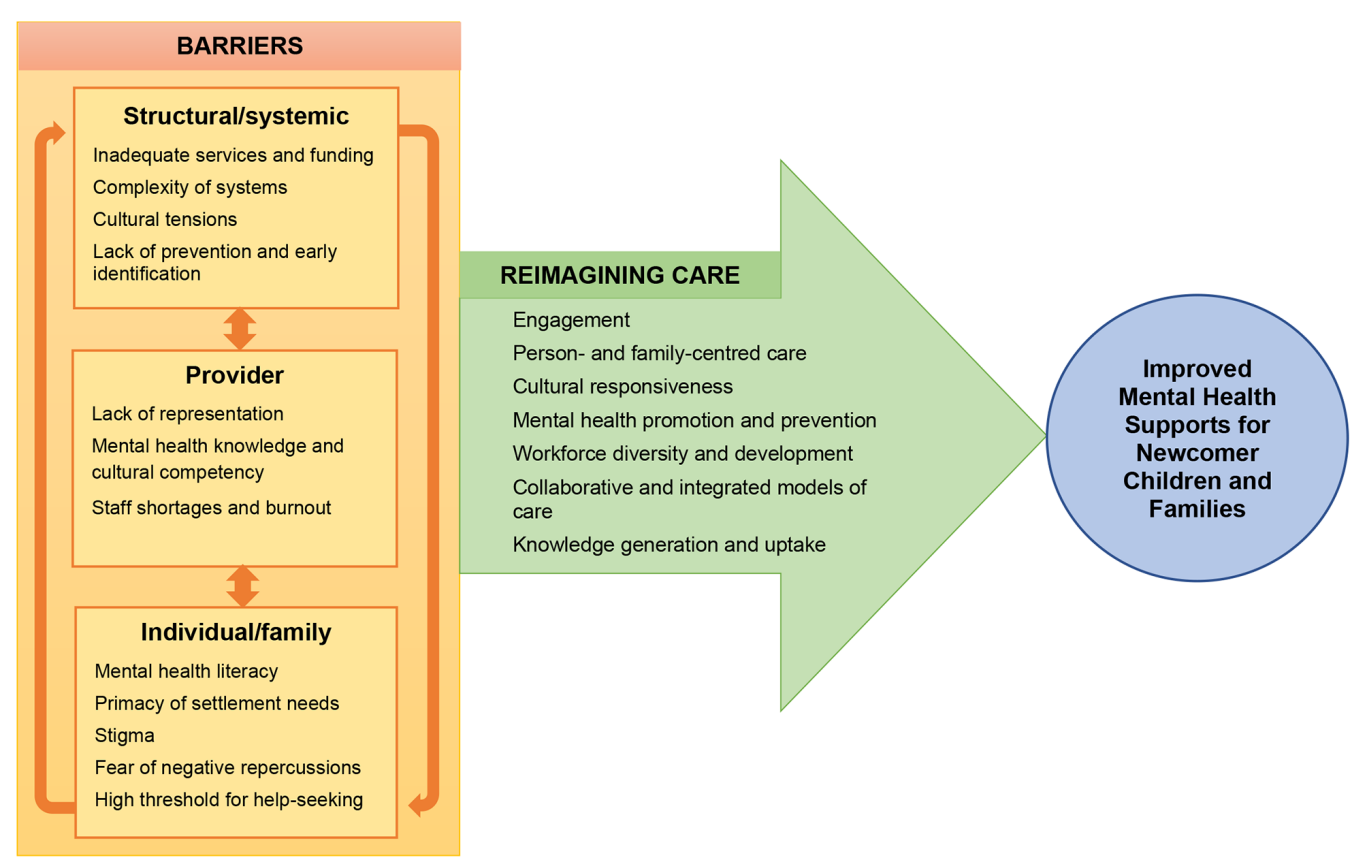
Barriers to accessing mental health supports and recommendations for reimagining care for newcomer children and families

Participants described multiple intersecting barriers that prevented newcomer children and families from accessing and benefiting from mental health supports, including barriers at the structural and systemic, provider, and individual and family levels. Participants also identified recommendations for addressing these barriers and reimagining mental health care for newcomer children and families. These themes and their sub-themes, as well as linkages among them, are summarized in Fig. 1 and described further below with supporting verbatim quotations provided in Supplementary Information. While the interview guide focused on Arabic-speaking newcomers, participants noted that their responses were relevant to all newcomers as well as members of racialized groups.

### Barriers

#### Structural and systemic barriers

Participants described intersecting structural and systemic barriers to accessing mental health care: inadequate services and funding; complexity of systems; cultural tensions; and, lack of prevention and early identification. There was consensus that existing mental health services were inadequate to meet the level of need, resulting in long wait times and delays in diagnosis and intervention. While these were universal challenges affecting timely access to care among the general population, participants observed that newcomer children and youth were disproportionately affected due to their unique language needs, cultural background, pre-migration and settlement experiences, and related mental health concerns. Providers outside the health sector noted that they were not typically funded to provide mental health programming for newcomer clients and the funding they did receive was limited and unpredictable. Participants further reflected on how newcomers’ mental health concerns typically emerged after the first one to two years in Canada, just as funding for settlement supports was typically discontinued. Constraints in level and allocation of funding had negative impacts on service providers’ ability to recruit and retain staff and provide consistent, needs-based services for newcomer children and families.

Participants described current service delivery systems as rigid, complex, and difficult to navigate, particularly for non-English speaking newcomers who lack familiarity with the Canadian healthcare system. Information and service delivery was described as scattered and disconnected, and often provided in ways that were not sensitive to newcomers’ unique circumstances. For example, several providers observed how common practices such as sending notifications about mental health care appointments or referrals through a mailed letter in English did not consider the issues of language and housing instability experienced by many newcomer families. Participants observed how issues such as families not responding to referrals or showing up for appointments were often interpreted as lack of engagement rather than indications of systems that were not responsive to the needs of newcomers.

Participants critiqued existing mental healthcare models and interventions as “Western-centric,” overly “clinical and medical,” and not relevant or adapted to the socio-cultural and religious background, migration history, and settlement experiences of newcomers. In particular, several providers highlighted the disconnect between the collectivist and family-oriented cultures from which many newcomers originate, and the individualistic and biomedical paradigm that predominates in the Canadian healthcare system. Participants observed that this disconnect resulted in newcomer families feeling misunderstood, disrespected, or even in conflict with one another when suggestions given by mental health professionals to youth were perceived by caregivers as culturally inappropriate. Language and interpretation issues exacerbated these challenges and contributed to a sense of frustration among newcomer children and youth as they struggled to discuss mental health concerns in English or through an interpreter. Participants noted the limited funding and availability of skilled interpreters and the added challenge of translating nuanced mental health concepts in an accurate and culturally appropriate way. Several providers also described instances of how for racialized newcomers in particular, these structural barriers intersected with systemic and institutional racism to foster distrust and disengagement from the mental health care system.

Participants noted a glaring absence of preventive mental health interventions for newcomer children and youth – as one provider stated: “It’s either psychoeducation or like, 18 weeks of cognitive behavioral therapy” (KI29, female, leadership role in mental health sector). The lack of prevention-focused interventions hampered early identification of child and youth mental health difficulties, resulting in newcomer families accessing services only when they were in crisis. Discussion of these challenges led to broader questioning around what constituted mental health support and whose role or mandate it was to provide such support to newcomer children and families. Some participants equated mental health support with clinical interventions such as psychotherapy delivered by a licensed mental health professional, explaining that their own lack of clinical training and concerns about liability made them hesitant to address mental health issues. In practice, however, participants acknowledged that providers such as settlement workers, childcare providers, educators, and family doctors often worked with newcomer families experiencing mental health challenges without having access to appropriate funding, expertise, or resources.

#### Provider-level barriers

Provider-level barriers related to lack of representation, mental health knowledge and cultural competency, and staff shortages and burnout. Participants observed that the sociodemographic characteristics of mental health care providers did not reflect those of newcomer children and families with most providers being white, female, English speaking only, and having no lived experience of migration. Lack of diversity and representation was noted as a systemic issue at the policy and leadership levels all the way through to frontline service provision. Several participants stated that Canadian educational and certification requirements (e.g., graduate or post-graduate degree) for delivering mental health services were barriers to hiring more diverse staff, thereby limiting availability of services in families’ native language and reducing trust and engagement with the mental health care system.

Participants explained how culture, language, stigma, and experiences of migration and acculturation resulted in mental health being conceptualized and expressed differently by newcomer families, often in ways that were not understood by providers. As one participant noted, newcomers use “a different language than the DSM-5” to talk about mental health (KI1, male, leadership role in settlement sector). Providers who were not mental health professionals, such as settlement and youth workers, further reflected on how their own lack of knowledge and training limited their ability to identify potential mental health concerns and make appropriate referrals, with some describing themselves as “helpless” and afraid to “do the wrong thing.”

Participants described how the general lack of cultural competency in all service delivery systems – not only mental health – meant that they were constantly pulled into the role of “middle person” between newcomers and other service providers, contributing to a high level of staff burnout. Burnout appeared to be particularly acute among providers who identified as racialized and/or had lived experience of migration. While these characteristics contributed to increased trust and rapport with newcomer clients, racialized and immigrant-background providers described bearing a greater workload burden due to a personal sense of obligation as well as the limited number of staff with language and cultural competency. As one provider noted, “racialized people in mainstream agencies tend to do double or triple the work because our work doesn’t go from 9 to 5” (KI58, female, frontline staff in youth development sector). Staff burnout was further exacerbated by inadequate staffing, large caseloads, and the prolonged personal and professional impacts of the COVID-19 pandemic.

#### Individual and family-level barriers

Participants identified several individual and family-level barriers related to mental health literacy, primacy of settlement needs, stigma, fear, and a high threshold for help-seeking that influenced newcomer families’ demand for and access to mental health support. Lack of mental health literacy related to recognition and perceptions of mental health symptoms and knowledge of available supports was seen as a major impediment to help-seeking. Participants noted that newcomers’ material, financial, and other basic needs often took precedence over their mental health, and caregivers had little time or “emotional energy” to recognize and address their own or their children’s mental health concerns. Previous negative experiences with the healthcare system was identified as an additional barrier.

Participants emphasized the deep-seated stigma and fear associated with disclosing mental health concerns. For many newcomers of Middle Eastern and other ethnocultural backgrounds, the concept of mental health was often equated with being “crazy” and having a psychiatric illness that required hospital or clinic-based care. Participants described fears expressed by newcomer children and caregivers around potential negative repercussions of disclosing a mental health concern, such as the reaction of their family or community, impacts on their immigration status, and the risk of children being removed by child protection services. Younger children’s access to care may be particularly constrained due to their reliance on caregivers as gatekeepers to services. Participants observed how these individual- and family-level barriers intersect to result in newcomer families accessing care only when the child or youth is already in crisis.

### Reimagining care

Participants’ recommendations for improving access to and quality of newcomer mental health care clustered around the following sub-themes: engagement; person- and family-centered care; cultural responsiveness; mental health promotion and prevention; workforce diversity and development; collaborative and integrated models of care; and, knowledge generation and uptake. Phrases such as “reimagining” care, “unlearning and relearning,” and “disrupting and dismantling the status quo” were used, indicative of a consensus that the current system was failing to address newcomers’ mental health needs. Providers noted that while “small pockets” of innovative practice existed, they were not “joined up” and progress was slow, patchwork-like, and incremental at best.

### Engagement

First, participants emphasized the need to move away from ad hoc and tokenistic attempts at newcomer engagement towards a more robust, planned, and integrated mechanism for amplifying and listening to newcomer voices. Participants noted that newcomer engagement around their needs and preferences for support should take place at all levels – from client feedback to co-creation of programs and services to representation at policy and program planning fora. Several providers described examples of successful newcomer engagement strategies, such as the formation of newcomer youth committees to co-design program offerings and working with ethnocultural organizations to reach underserved groups. Respondents also noted, however, that truly impactful engagement was time and resource-intensive, and that most mainstream organizations were not designed or resourced to meaningfully integrate newcomer voices into program design and delivery.

#### Person- and family-centred care

Second, person- and family-centered and community-based care was seen as essential to improving access to and quality of mental health supports for newcomer children and families. The phrase “meeting people where they’re at” was frequently used as a metaphor for a fundamental shift in the model of care whereby systems and services adapt to the needs, priorities, and preferences of newcomer communities rather than the other way around. Participants described the need to meet newcomers where they are *physically* – in terms of where newcomers live, learn, and receive services – as well as *psychologically* – in terms of where they feel safe and a sense of trust and belonging. Embedding mental health supports in community settings such as schools and ethnocultural associations could reduce transportation barriers as well as alleviate stigma and mistrust by locating services in spaces where newcomer children and families feel safe with trusted providers or community members. Participants also highlighted the need for systems and services to embrace a family-centered approach to mental health given the collectivist and family-oriented societies from which many newcomers originate. For example, providers noted the importance of sensitively engaging parents in identifying and responding to newcomer children’s mental health needs to ensure that care does not clash with cultural values, thereby causing or exacerbating intergenerational conflict.

#### Cultural responsiveness

Third, participants highlighted the importance of cultural responsiveness, which they described as involving three interrelated components: first, practicing cultural humility and acknowledging the biases inherent in one’s own training, knowledge, and background; second, learning how cultural and religious background and the migration experience shape different conceptualizations and expressions of mental health; and third, learning to speak the “language of mental health” in a way that resonates with families from different ethnocultural and religious backgrounds. Some providers, for example, described using words such as “stress” or “pressure” to discuss mental health concerns rather than depression or anxiety, which in their experience had more negative connotations among newcomers. Other recommendations included more thoughtful and rigorous cultural adaptation of evidence-based mental health interventions to reflect newcomers’ lived experience, which may include experiences of trauma and displacement-related adversity. Some providers also suggested a “champion model” in which community members act as cultural brokers or interpreters who help newcomer families bridge communication gaps and navigate cultural differences. Several participants noted the untapped potential for ethnocultural organizations to play this role and called for greater funding and engagement of these small, typically informal groups by mainstream service providers.

#### Mental health promotion and prevention

Fourth, participants highlighted the need for greater investments in mental health promotion and prevention as part of the continuum of mental health supports for newcomer children and families. More focus on prevention could help to bolster protective resources, mitigate the effects of risk factors, and improve early identification and intervention, thereby relieving pressure on tertiary mental health services. Participants also described the need for a more holistic approach to mental health, with greater attention to mental health promotion and awareness raising in families, schools, and communities to normalize discussions of mental health and help-seeking. Youth-focused providers noted that overtly mental health-focused interventions can be stigmatizing and intimidating for children and youth, emphasizing the importance of engagement and trust-building through recreational activities such as sports or creative arts. Creation of more youth-friendly “safe spaces” would provide an entry point for newcomer children and youth to discuss mental health issues and be connected to further support if needed. Participants recommended building capacity for “mainstreaming” mental health promotion and prevention activities as integral components of all programs and services that serve newcomer families – regardless of sector – as well as piloting preventive interventions that are tailored to the strengths and challenges of newcomer families and communities.

#### Workforce diversity and development

Fifth, participants discussed the need to “change the face” of mental health care by hiring and retaining a more racially and culturally diverse workforce that better reflects newcomer communities. In addition, they recommended more learning and peer support opportunities for staff – particularly those working in non-health sectors – to increase their cultural competency and ability to identify and refer newcomers with mental health concerns to available supports. Some providers went further to state that it was “time to decentralize” the workforce and train “different types of mental health workers” (KI18, male, leadership role in youth development sector). Specific suggestions included the mobilization of peer support workers to enhance service delivery and reach, thereby leveraging existing knowledge, language skills, and trust to support their own communities. Several participants, however, cautioned that mental health was a regulated profession to ensure duty of care and client safety, and emphasized the need for clearly defined roles and appropriate training, supervision, and accountability structures.

#### Collaborative and integrated care

Sixth, participants advocated for new models of collaborative and integrated care to improve service access and address social determinants of newcomer mental health [24]. Specific suggestions included a holistic and multi-sectoral approach in which mental health services are embedded and co-located with settlement and other social services to simplify service navigation as well as address material and social conditions that may increase risk of poor mental health. Participants noted that this integrated approach to service delivery would require a shift from existing referral and coordination mechanisms towards more collaborative, cross-sectoral, and interdisciplinary models of care. Some providers, however, also expressed concerns around potential ‘role creep’ and ensuring that mental health professionals were not being diverted away from clinical work to provide case management or settlement services.

#### Knowledge generation and uptake

Finally, participants called for more effective knowledge generation and uptake to inform improvements in program design, implementation, and evaluation. Participants noted the need for systematic and long-term data collection and monitoring to identify patterns in newcomer mental health and service utilization that could inform service delivery and planning. As noted above, cultural considerations should be embedded in research and evaluation activities, particularly when adapting and contextualizing mental health assessments. Participants further emphasized the importance of translational and applied research so that findings can be communicated back to provider and newcomer communities to co-develop, test, and scale up interventions. Several providers also noted the need for more concerted efforts to learn from innovative practices in other parts of Canada and around the world, and to partner with researchers and newcomer communities to co-create and pilot new models of care.

## Discussion

Findings from this study demonstrate how barriers at the systems, provider, and individual and family levels intersect to create disparities in access to and quality of mental health care for newcomer children and families. Similar findings published over a decade ago, including a Mental Health Commission of Canada (MHCC) report on mental health services for immigrant, refugee, ethnocultural and racialized groups, underscore the intractable nature of many of these challenges [15, 17]. Overall, participants in our study expressed a deep desire for systemic change, with most emphasizing supply-side structural and provider-level barriers and recommendations rather than individual demand-side factors. This perspective resonates with wider calls to shift from individual and group culture-based frameworks towards greater consideration of how structural and systemic factors produce and perpetuate inequities in newcomer mental health [25, 26].

Study findings suggest several ways in which mental health care for newcomer children and families could be “reimagined.” Participants advocated for a community-based, collaborative, and integrated model of care in which services to address material and social stressors – as well as mental health concerns – could be co-located and coordinated by a multidisciplinary team of providers. Embedding mental health supports with other social services and moving them from clinical to community settings were seen as vital strategies for increasing accessibility and reducing stigma and mistrust. These recommendations align well with Canada’s Mental Health Strategy [16] and the movement towards collaborative mental health care more broadly [27], and may be particularly relevant to newcomer families who experience intersecting stressors and barriers to care. Study findings also highlight significant gaps in the continuum of mental health supports for newcomer families, with many participants emphasizing the need for improved prevention and early intervention to reach children and families *before* they are in crisis [28]. Stepped care models, in which interventions are stepped up or down in intensity based on level of need, could be a useful framework for unifying mental health promotion, prevention, and treatment interventions across multiple sectors and providers [29]. Significant realignment of resources and the way in which services are organized and delivered, alongside rigorous evaluation, would be required to assess feasibility and effectiveness of these models of care for newcomer families and service providers.

Provision of programs and services along the full continuum of mental health supports is currently constrained by the lack of providers with appropriate language skills, cultural competency, and lived experience. Peer providers are a potential solution to this gap in the workforce, particularly for scaling up mental health promotion and prevention interventions. Peer providers, also known as lay or non-specialist providers, refer to individuals who have not received degrees in a mental health field (e.g., social work, counselling) or allied health profession (e.g., nursing) and who generally come from the same community as the population they serve [30]. There is now accumulating evidence from the United States and low- and middle-income countries that peer provider-supported or delivered mental health interventions are both feasible and effective at reducing disparities in access to care for underserved communities [31–33]. However, evidence of the feasibility and effectiveness of peer provider models in the Canadian context is scarce and disputed [34–36], and integration into the mental health care system has been uneven [37]. Expanding the mental health workforce by leveraging the resources of newcomer communities is a potential way to boost prevention and early intervention efforts, thereby enabling mental health professionals to focus on individuals with higher levels of clinical severity.

### Strengths and limitations

Study strengths include engagement with a study advisory group composed of multi-sectoral service providers to inform research questions, study design, and interpretation of findings. We were able to recruit a diverse sample of leadership and frontline staff from service providers across a range of sectors including community, ethnocultural, and religious organizations, and over half of the study participants came from an immigrant and/or racialized background. Participants responded to the interview guide in relation to newcomers from different ethnocultural and language groups rather than Arabic-speaking newcomers specifically as originally intended. Hence, study findings highlight principles and best practices that are broadly applicable to newcomer children and families from diverse ethnocultural backgrounds. The study would have benefited from greater representation from the education sector given the significant role that schools play in identifying and responding to child and youth mental health concerns. Finally, findings are based on respondents’ experiences in Hamilton specifically and may not be applicable to other Canadian contexts, although general consistency with results from other research suggests broader relevance of study findings and implications [12].

## Conclusions

Study findings suggest the need for a paradigm shift in the way that mental health care is conceptualized and delivered to newcomer children and families in Canada. Participants called for greater structural *and* cultural competency in the mental health care system as well as a public health approach that prioritizes mental health promotion and prevention alongside treatment. As noted in previous research [15, 16], new models of collaborative, community-based, and newcomer-centred care are urgently needed to close the gap in mental health care access and contribute to the well-being and resilience of new Canadians.

### Electronic supplementary material

Below is the link to the electronic supplementary material.

Supplementary Material 1

## Data Availability

The data that support the findings of this study are not publicly available due to the inclusion of information that could compromise participant privacy but are available from the corresponding author on reasonable request.
