# Supplementary material for: Reimagining mental health care for newcomer children and families: a qualitative framework analysis of service provider perspectives
Source: BMC Health Serv Res. 2023 Jun 27;23:699. doi: 10.1186/s12913-023-09682-3 (PMC10303766; doi:10.1186/s12913-023-09682-3)
Supplement: Supplementary file 1 — Supplementary Material 1 [file 12913_2023_9682_MOESM1_ESM.docx]

**Supplementary Material.** Illustrative quotations from providers

| **Categories** | **Themes** | **Quote** |
| --- | --- | --- |
| **Structural and systemic barriers** | Inadequate services and funding | “If you want the honest truth, I think there's a huge gap. Even from the [program name] perspective, if you ask me, are we doing everything possible? Unfortunately, we're not. And the challenge is resources. It's not the lack of willingness to do it, it is lack of funding, lack of resources. And unfortunately, lack of focus as well.” (KI 7, female, leadership role in settlement sector) |
|  |  | “In fact, we have had consultations with service providers in the past few years, even funded by IRCC. And mental health supports always come up as an area where there's a gap, where there needs to be funding and more support.” (KI 15, female, leadership role in settlement sector) |
|  | Complexity of systems | “And then when we talk about the system of care for mental health services, I mean, I'm going to tell you stuff you already know, it's little pockets of funding here, there and everywhere that work really well to try and coordinate and collaborate and create pathways to make it easier. But that system is a fractured system. And I can appreciate as somebody who's new, speaks a different language, has a different cultural understanding, how overwhelming that system could be to navigate. When you're dealing with an issue, you know, like dealing with the issue can be enough to fill your cup, if you then are having to climb mountains to get access, and you have language barriers, it could just be insurmountable and really overwhelming for families.” (KI 13, female, leadership role in public/government sector) |
|  | Cultural tensions | “Yeah, I would say they're not culturally appropriate. Like, it's very, like clinical and medical. And I think that's probably why the youth that I went with to her appointments was like, I don't want any part of this, because it was like a psychiatrist who had probably never worked with an interpreter before. And like, it was very clinical…And so I think they're just not culturally appropriate, not necessarily trauma informed, like I'm sure the psychiatrists here are used to, you know, the traumas that people would experience here, but not traumas in the sense of like, war, displacement, leaving your culture behind. And also just working with people who come from a place where mental health is stigmatized.” (KI 55, female, frontline role in settlement sector) |
|  | Lack of prevention and early identification | “There's not much in the way of prevention…we would hope that that youth would get to us kind of early days, and maybe could do a bit of talk therapy and, you know, bolster some of those supports, and maybe connect them with some rec or you know leisure or something that makes their quality of life better. But oftentimes, it's like, you have missed so much school now that the school has called the Children's Aid Society, and now they're involved…” (KI 29, female, leadership role in mental health sector) |
|  |  | “But I think so much of what we see that ends up as a referral to a tertiary level service could have been avoided if somebody had really, you know, paid attention and listened to what the need is, and put those supports in place at the very beginning.” (KI 13, female, leadership role in public/government sector). |
| **Provider-level barriers** | Lack of representation | “There are services that we can refer people to that are, for example, there are mental healthcare services that are targeting immigrant kids and teenagers. But I find that a lot of times, some of them bounce back. And sometimes that may have to do with language, sometimes may not have to do with language because they do have cultural interpreters. I think there is a lack of professionals in the field that are representative of those communities. I think people can speak more freely when they speak in their own language, when they are talking to people who have some knowledge of their culture. But when that piece is absent, people lose interest, parents lose interest and children lose interest.” (KI 61, female, frontline role in youth development sector) |
|  | Mental health knowledge and cultural competency | “I think number one would be to have this information. Even now if I'm thinking about myself and our staff, like to be familiar with what to look for, to have this information for us. Because sometimes we may not see this as a mental health problem…we are feeling like helpless, because we don't know how to help, we don't know what to do, how to handle and not to do wrong things.” (KI 28, female, leadership role in settlement sector) |
|  |  | “If they don't understand the religious and the cultural foundation, concepts, mindsets – a lot of times what happens is that leads to a clash within the family, especially for seeking help for a youth. The parents would feel that okay, now somebody is telling my child to rebel, because it doesn't match with their cultural norms and practices.” (KI 7, female, leadership role in settlement sector) |
|  | Staff shortages and burnout | “Like there are two Arabic-speaking SWIS^1^ workers for the whole city of Hamilton. So you can't ask them to be available at 20 different schools to do this kind of work.” (KI 66, female, frontline role in education sector) |
|  |  | “I find that it is racialized people in mainstream agencies that end up being overburdened and overworked. And the expectations are that we can always give more, you know, and we have that moral responsibility towards other people, especially those people that we can identify with, that we speak the language with.” (KI 61, female, frontline role in youth development sector) |
| **Individual and family-level barriers** | Mental health literacy | “Mental health needs are there but they don't really see it, like youth don't really see it themselves or they don't really see it as an issue.” (KI 51, female, frontline role in settlement sector) |
|  | Primacy of settlement needs | “They are thinking about essential things, food, education, employment, you know. Mental health for them comes way after, doesn't come at the beginning. So, they are dealing with it, they are dealing with poor mental health, but it is not a priority because they are trying to survive their daily basic lives.” (KI 50, female, frontline role in settlement sector) |
|  | Stigma | “There's a word in Arabic where it's like, I'm mentally ill. What's my family gonna do? Oh, should I tell my mom right? Should I not tell my parents? They're scared of what their family would say because it's so frowned upon.” (KI 52, female, frontline role in settlement sector) |
|  | Fear of negative repercussions | “So they wouldn't share certain information with us to say that they're having difficulties with the children because they think that we're gonna call Children Aid Society and we're gonna take the kids away from them.” (KI 56, female, frontline role in settlement sector) |
|  | Help-seeking threshold | “Sometimes they seek help after it's reached a point that is really, really bad.” (KI 54, female, frontline role in settlement sector) |
| **Reimagining care** | Engagement | “It's really important to hear from and give opportunities for families to be part of the development of whatever it is, services or supports they're accessing, and finding ways to make their voices be heard in terms of what they're receiving.” (KI 25, female, leadership role in mental health sector) |
|  | Person- and family-centred care | “So how do we rearrange ourselves to make sure that we're able to meet them where they're at and deliver that service in a way that is comfortable for them? Be more deliberate and strategic about meeting them where they're at and thinking about how do we deliver it, create a model that doesn't exist right now and help meet their needs in a different way.” (KI 14, female, leadership role in public/government sector) |
|  |  | “Family needs to be involved, this idea of this person is an adult. And until they give us permission, we cannot share their information. Unless that person is going to be living by themselves in a bubble by themselves and not interacting with family, family should be involved in their care taking into consideration people's cultural backgrounds and religious beliefs. They are big, big in our cultural backgrounds and religious beliefs shape who we are. If we have been raised with those beliefs, they shape who we are. So that also should be put into context when coming up with a care plan for someone that has mental health related issues.” (KI 27, female, leadership role in settlement sector) |
|  | Culturally responsiveness | “So not taking their behaviors that are considered acting out as like indicative of something wrong with them. But understanding where that's coming from, in a very non-judgmental and empathetic way. And I think it's really important also within anti-oppressive practice, not to center like our understandings of what a life should look like, what somebody's values are, but not assuming that our understanding of mental health is even superior to theirs. So when we say people have stigma, because they don't agree with ideas of depression and anxiety, or whatever, why do we think that there's something like that they're stupid for disagreeing? No, they have a reason for that. The DSM is a very – it's a product of our culture. So it's natural that people would disagree and have different interpretations of it…But clearly, the idea of mental illness is not universal. So not trying to hold people to our standards and meeting them where they're at.” (KI 64, female, frontline role in ethnocultural sector) |
|  |  | “I think the first key principle should be cultural relevancy. Period, like I, if you don't have it, if you're a mental health agency, you are under obligation to make sure you have it. Because the issue of mental health is extremely personal, and vital to people's way of life. So if I come from a certain culture, which is my way of life, traditions, and everything, and you want to talk about, talk to me about something that's sensitive, in particular, that evokes all kinds of cultural and traditional stuff, if you don't have context for what you're talking, you've lost me. Zero, I'm not paying attention to that because I think you are attacking my culture, I think you’re attacking me. But if you have context, even if I want to disagree with you, I still listen, because I know you know what you're talking about and you understand why I'm not doing certain things, but you think it's a different way to actually do it. So I think cultural relevance is number one.” (KI 18, male, leadership role in youth development sector) |
|  | Mental health promotion and prevention | “It's the mental wellness, it's the health promotion, like those are the areas that if we could get kind of more supports in those areas, that would help take the pressure off of the more complex clinical needs that we have in our community, and hopefully address them kind of at an early intervention, prevention level.” (KI 5, female, leadership role in mental health sector) |
|  | Workforce diversity and development | “So, I mean, yeah, having a team of clinicians who speak different languages, who are from different backgrounds…having a team that really is reflective of the community that we serve…Training, absolutely. I think that there's always ongoing learning that can happen, working with this population. So yeah, having access to regular ongoing training, establishing, as I said before, a network so that…you have a better understanding of what each person is doing and you can sort of consult with folks.” (KI 25, female, leadership role in mental health sector) |
|  |  | “Because getting training is so expensive and honestly, inaccessible, that when a certain class of people in certain races are accessing, it is time to decentralize that stuff. Because the moment you start to provide culturally relevant mental health services, then that obligates you to start training different types of mental health workers, so the two go hand in hand.” (KI 18, male, leadership role in youth development sector) |
|  | Collaborative and integrated models of care | “I think the best practice is for the settlement and health agencies to partner together in order to have a program right now. I don't think most of the settlement agencies have the capacity to run a program like this, a specialized program like that from the clinical lens. And then the health agencies don’t have the capacity in terms of cultural piece, because they have their own interpretation of mental health that might not be defined in an Arabic culture. So I think there should be a different layered program, combine these two agencies together for one program if that makes sense.” (KI 1, male, leadership role in settlement sector) |
|  | Knowledge generation and uptake | “We do have an obligation to try and pilot new things. Absolutely. Because if we wait for the system to change, I think we're more effective in piloting, trialing new things, having the proof and then, you know, lobbying efforts. I don't think we can wait for the system to change because of the bureaucratic wheels and the political landscape and the cycle of municipal and provincial elections.” (KI 13, female, leadership role in public/government sector) |

^1^ Settlement Workers in Schools
